# Physical and Electrical Characterization of Synthesized Millimeter Size Single Crystal Graphene, Using Controlled Bubbling Transfer

**DOI:** 10.3390/nano11102528

**Published:** 2021-09-27

**Authors:** Soukaina Ben Salk, Reetu Raj Pandey, Phi H. Q. Pham, Di Zhou, Wei Wei, Guillaume Cochez, Dominique Vignaud, Emiliano Pallecchi, Peter J. Burke, Henri Happy

**Affiliations:** 1 University of Lille, CNRS, UMR 8520—IEMN—Institut d’Electronique de Microélectronique et de Nanotechnologie, F-59000 Lille, France; Soukaina.ben-salk@univ-lille.fr (S.B.S.); reetu-raj.panday@univ-lille.fr (R.R.P.); di.zhou@univ-lille.fr (D.Z.); wei.wei@univ-lille.fr (W.W.); guillaume.cochez@univ-lille.fr (G.C.); dominique.vignaud@univ-lille.fr (D.V.); 2 Department of Electrical Engineering and Computer Science, University of California, Irvine, CA 92697, USA; PhiP1@uci.edu (P.H.Q.P.); pburke@uci.edu (P.J.B.)

**Keywords:** monocrystalline graphene, electrochemical delamination, bubble-free transfer, Raman spectroscopy, defect-free, electrical characterization

## Abstract

In this work, we have investigated the influence of the transfer process on the monocrystalline graphene in terms of quality, morphology and electrical properties by analyzing the data obtained from optical microscopy, scanning electron microscopy, Raman spectroscopy and electrical characterizations. The influence of Cu oxidation on graphene prior to the transfer is also discussed. Our results show that the controlled bubbling electrochemical delamination transfer is an easy and fast transfer technique suitable for transferring large single crystals graphene without degrading the graphene quality. Moreover, Raman spectroscopy investigation reveals that the Cu surface oxidation modifies the strain of the graphene film. We have observed that graphene laying on unoxidized Cu is subject to a biaxial strain in compression, while graphene on Cu oxide is subject to a biaxial strain in tension. However, after graphene was transferred to a host substrate, these strain effects were strongly reduced, leaving a homogeneous graphene on the substrate. The transferred single crystal graphene on silicon oxide substrate was used to fabricate transmission line method (TLM) devices. Electrical measurements show low contact resistance ~150 Ω·µm, which confirms the homogeneity and high quality of transferred graphene.

## 1. Introduction

The extraordinary properties of graphene has garnered much attention, and has motivated researchers to investigate its technological applications due to its high electrical and thermal conductivities, flexibility and stability [1,2,3,4,5,6,7]. Chemical vapor deposition (CVD) on Cu has been broadly used to synthesize large-area and high-quality graphene [8,9,10,11]. Recently, the synthesis of large-area monocrystalline graphene has been of great interest due to the lack of grain boundaries which are responsible for graphene performance degradation. Because the majority of applications require the as-grown graphene to be transferred to a host substrate, it is important to study how large monocrystalline domains behave during this process. Indeed, the transfer of graphene can have a considerable impact on its physical and electrical properties [12,13,14,15,16,17,18]. Transfer steps may damage the graphene film, causing cracks, wrinkles and polymer residues and thus, reducing the carrier mobility of graphene, a parameter of the upmost importance for electronic applications [19]. Several methods have been developed to transfer the graphene from metal to the host substrates. The most common transfer technique involves the wet etching of the metal layer used as a catalyst template for the graphene growth, typically Cu for monolayer graphene. This technique is reliable and well-studied [9,20,21], but has the disadvantage of a slow etching process. Furthermore, destroying the Cu film is not environmentally friendly. To overcome these limitations, remarkable progress has been made in the development of transfer techniques that avoid the etching of growth substrate. The most used method is the so-called electrochemical delamination transfer technique [16,22,23,24,25,26,27]. Using this technique, minutes are sufficient to separate the graphene layer from the growth substrate, compared to many hours required by the wet etching transfer method. Furthermore, this transfer technique significantly reduces metal consumption and the associated environmental pollution, and allows for the reuse of the metal substrate, [16,22] which is especially promising for large-scale, low-cost production.

In this study, large isolated graphene monocrystals of millimeter scale dimensions (~5 mm) have been synthesized on polycrystalline Cu foil [13]. After the growth, the graphene-covered Cu films were annealed to promote Cu oxidation. The oxidation enables the ability to easily choose the sample to be used for transfer, as with the Cu oxidation, the crystalline graphene samples become visible by eyes with the Cu oxidation. Afterwards, monocrystals of graphene, with and without post-growth oxidation, were transferred to Si/SiO_2_ substrate using an optimized electrochemical delamination method as presented in the Supporting Information. SEM and optical microscopy were used to analyze the morphology and quality of the material before and after transfer. Micro-Raman spectroscopy has been used to investigate how the oxidation of the Cu underneath the graphene affects the graphene properties, by studying the strain and doping of the graphene films. Finally, transmission line measurement (TLM) structures were fabricated using the transferred graphene. Measurements performed on six TLM patterns, fabricated on a graphene single crystal, reveals a relatively low value of the contact resistance (~150 Ω·µm) which is attributed to the good quality of the transferred graphene and the fabrication process. The results presented in this work provide a path towards large-scale high-quality monocrystalline graphene transfer, which could contribute to the development of graphene-based electronic devices.

## 2. Experimental

In this section, we present the detailed analysis of the bubble-free transfer process on the morphology and on the physical and electrical properties of the synthesized monocrystal of graphene.

### 2.1. Growth of Large-Size Single Crystal Graphene

The synthesis of millimeter size monocrystalline graphene—first reported in Ref. [13]—begins with an electrochemical polishing of the Cu to improve the surface quality and thus decrease the nucleation density. The crystal growth is carried out inside enclosed Cu pockets, to further reduce the nucleation rate. The growth consists of two phases: a low methane flow is first employed to induce a low nucleus density, followed by an increase in methane flow in order to increase the size of the crystals without needing extended growth durations, as schematized in Figure S1 in the Supporting Information. After CVD growth, the Cu pocket is unfolded and the as-grown sample (Figure S2a) is exposed to an intense flash light to identify the graphene crystals with naked eye (Figure S2b). The samples are then annealed on a hot plate (250 °C for 15 min) in order to oxidize the Cu surface and to improve the visual contrast of graphene monocrystals which can be clearly seen, with a typical size of ~5 mm (see Figure S3).

### 2.2. Electrochemical Delamination Transfer Process of Graphene (Controlled Bubbling Transfer)

Firstly, thin layer of 5% PMMA (polymethyl methacrylate) diluted in anisole was spin-coated onto graphene on one side of the Cu foil (3000 rpm, 1000 rpm/s, 10 s). Next, the sample (PMMA/graphene/Cu) was cured at 90 °C for 30 min with very slow heating and cooling rates (30 min), in order to prevent cracks in graphene caused by the strain induced in PMMA resist. Because the graphene was grown on both sides of the Cu foil, the graphene on the back-side was removed by O_2_ plasma etching (2 min, 50 W RF power) before being subjected to delamination.

For electrochemical delamination “controlled bubbling transfer”, PMMA/graphene/Cu stacks were immersed gradually in KOH solution (40 mmol/L) and a two-electrode system held at a potential of 2.7 V. A continuous voltage was then applied between the cathode (the polymer/graphene/Cu sample) and anode (glassy carbon rod) in an electrolytic cell. KOH was used as the electrolyte solution and the decomposition of H_2_O causes the generation of H_2_ bubbles at the Cu-graphene interface:

2H_2_O (l) → H_2_ (g) + 2OH

H_2_ bubbles help separate graphene from the Cu surface. However, the uncontrolled production of hydrogen bubbles can cause mechanical damage in the graphene film. Complete delamination occurs within a few minutes (≤20 min). The graphene/PMMA film was then thoroughly rinsed (10 times) in deionized water to ensure the removal of residual KOH.

Finally, the graphene/PMMA film was transferred to Si/SiO_2_ substrate and dried at 90 °C for 30 min with very slow heating and cooling rates (30 min) in order to remove the water trapped underneath the graphene. The complete delamination and transfer process of graphene is presented in the Supporting Information Figures S4 and S5 and the detailed optimization of the electrochemical delamination “controlled bubbling transfer” process is given in the Supporting Information Figure S6. Figure S6II shows the PMMA/graphene film floating on the surface of the solution after separation from copper foil (Figure S6IIa) without the presence of bubbles trapped under the film. The same film is shown just after transfer on SiO_2_ (Figure S6IIc) and after annealing (Figure S6IId). The film is homogeneous without any trapped bubbles at the interface. Scanning electron microscopy (SEM) images of the samples were taken with a Zeiss Ultra 55 SEM. Raman spectrum were obtained with a confocal micro-Raman LabRam HR spectrometer (Horiba Jobin-Yvon) using an excitation laser (473 nm) focused with 100× objective.

## 3. Results

In this section, we present the detailed analysis of the controlled bubbling transfer process on the morphology, physical and electrical properties of the synthesized monocrystal of graphene.

### 3.1. Morphological and Structural Characterization of the As-Grown Single-Crystal Graphene

**Figure 1:**
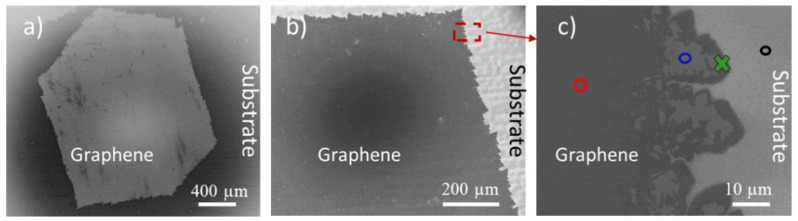
Graphene monocrystal with post-growth annealing before transfer, (**a**) SEM image of a hexagonal monocrystal (**b**) edge of the graphene monocrystal, (**c**) magnified area framed in red in panel b, showing one graphene edge close to which Raman spectra were measured (colored marks).

Before the transfer process, the synthesized graphene crystals were fully characterized using SEM and Raman spectroscopy as they can provide the local information on the structural uniformity and homogeneity of graphene samples. The SEM allows imaging peculiarities of graphene, for example the shape of the synthesized crystals, grain boundaries and the presence of holes and discontinuities in the sheet of graphene. Figure 1a is an inverted contrast SEM image of a graphene sample after post-growth annealing in air. The hexagonal shape and the lack of observable grain boundaries suggest that the synthesized graphene is monocrystalline, which is confirmed by selected area electron diffraction (SAED) as reported in [13].

**Figure 2:**
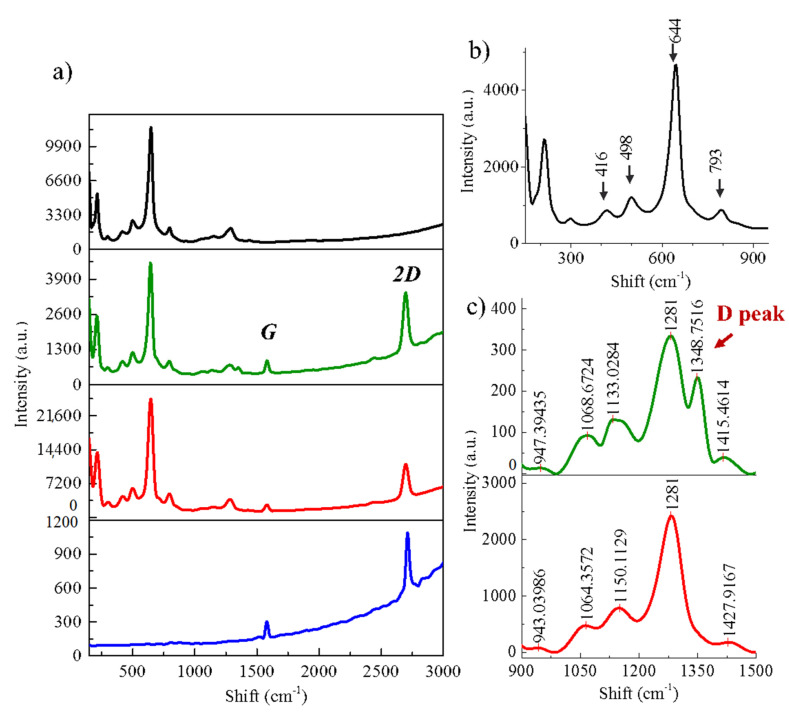
(**a**) Raman spectra corresponding to areas pointed in Figure 1.c using the same colors, (**b**) zoom-in between 0 and 900 cm^−1^ of the black spectrum showing Cu oxide peaks and (**c**) zoom-in between 900 cm^−1^ and 1500 cm^−1^ for the red and green spectra of Figure 2a.

Figure 1c is a magnification of the area framed in Figure 1b, where we identify the three different contrasts which are pointed out by red, blue and black circles. The black circle refers to the oxidized Cu substrate—free of graphene—and the red and blue circles correspond to the areas covered by the graphene film. In order to determine the source of the contrasts presented on the surface of the hexagonal domain, the areas marked by black, blue and red circles, and the edge of the graphene crystal (indicated by a green cross) have been studied by Raman spectroscopy. Figure 2a presents the typical Raman spectra corresponding to the different areas pointed in the SEM image of the Figure 1c with the respective colors. As expected, graphene peaks (G at ~1580 cm^−1^ and 2D at ~2700 cm^−^^1^) are present in all areas except the black ones corresponding to the substrate. The blue spectrum in Figure 2a is the typical spectrum of a high-quality graphene monolayer on Cu with I_2D_/I_G_ ~ 2. The red and green spectra also feature the G and 2D peaks, but have extra peaks between 900 cm^−^^1^ and 1500 cm^−^^1^ as represented in Figure 2c. It should be noticed that in the case of the red area, none of these peaks correspond to the D peak (1349 cm^−^^1^), a signature of structural defects in the graphene. The D peak is only observed at the edge of graphene (green cross in SEM image of the Figure 2a).

The absence of the D peak away from the edge of graphene as well as the presence of G and 2D peaks in areas pointed by the red and blue circles with an intensity ratio I_2D_/I_G_ > 2 indicates that the graphene crystal is monolayer, homogeneous and of high quality. In Figure 2b, peaks below 900 cm^−^^1^ are attributed to Cu oxides (CuO and Cu_2_O) [28,29]. These Cu oxide peaks appear in the red and green spectra in Figure 2a and are absent in the blue area. These oxides are therefore the source of the contrast observed in the different areas of the graphene monolayer. The contrast is observed over the entire surface covered by graphene showing that the oxidation under graphene is not homogeneous. We can thus consider that the graphene crystal lays on two different substrates, Cu and Cu oxides.

### 3.2. Morphological Characterization of Single-Crystal Graphene on SiO_2_ after Transfer

**Figure 3:**
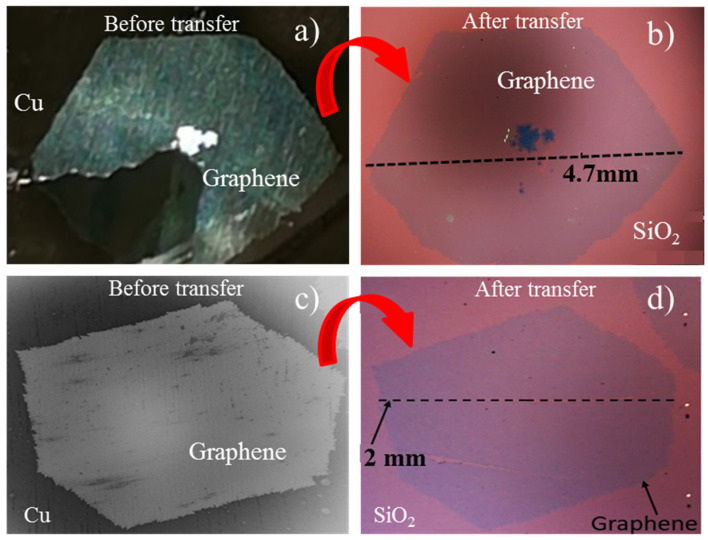
Optical (**a**,**b**,**d**) and SEM (**c**) images of single crystal graphene before and after transfer to SiO_2_ substrate. (**a**,**b**) The sample without any annealing. (**c**,**d**) The sample with post-growth annealing.

**Figure 4:**
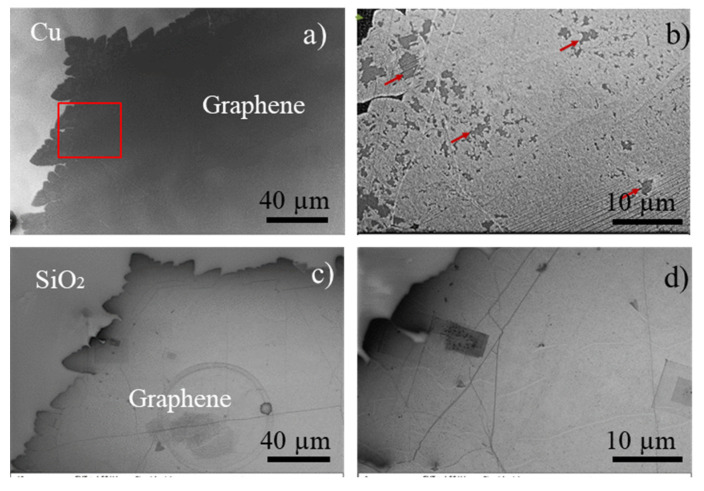
SEM images of graphene samples with post growth annealing (**a**,**b**) before transfer, (**b**) magnification of red square in panel a, red arrows pointing the unoxydized Cu areas, (**c**,**d**) same area after transfer, the graphene is homogeneous.

The electrochemical delamination method described above was used to transfer the isolated large crystal graphene samples with and without air post-growth annealing. The optical images in Figure 3 show the same graphene single crystals before and after transfer, confirming that the electrochemical transfer yields a homogeneous layer of graphene with large areas (mm-scale) without tears or holes. This also demonstrates that the transfer process maintains the structure of graphene and should be achieved without damage over mm-scale areas. We do note that during transfer, there can be damage to the graphene film from human handling of the samples (as observed in the Figure 3d), but the majority of the area of the transferred sample remains available for device fabrication on mm-scale. To further analyze the oxidation effects of graphene crystals after post growth annealing, SEM investigations were carried out as shown in Figure 4. Before transfer, the Cu oxidation under graphene is nonhomogeneous as pointed out by the red arrows in Figure 4b which show areas where graphene stands on unoxidized Cu. However, after the graphene transfer of this crystal, the surface of graphene is uniform, keeping its original shape with less visible damage as seen in Figure 4c,d. The copper crystal boundaries are also visible. This confirms that the transfer process can be carried out even with a partial oxidation of the Cu surface. The graphene layer after transfer remains homogeneous even though the oxydized Cu layer below the graphene was not. Analysis of the optical and SEM images shows that the controlled bubbling transfer electrochemical delamination transfer process provides a homogeneous layer of graphene.

### 3.3. Characterization by Raman Spectroscopy

**Figure 5:**
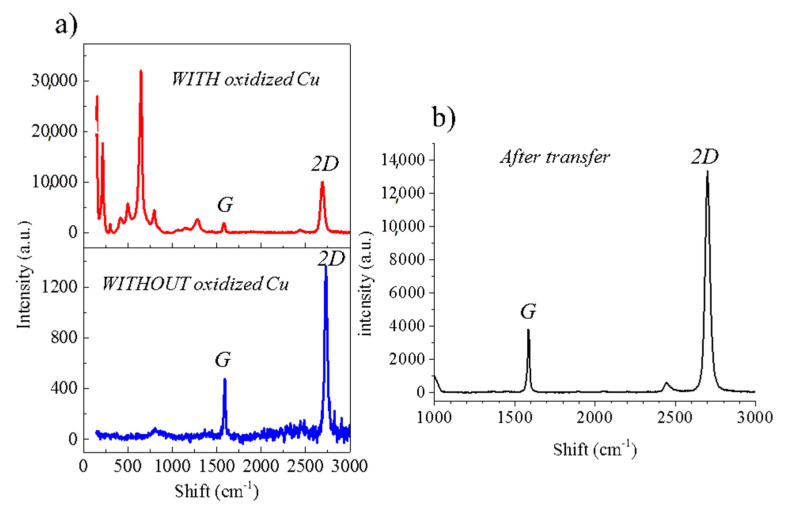
Raman spectra of graphene from Figure 1c after subtracting the baseline. (**a**) Red represents an area where graphene is on Cu oxide, blue represents an area where graphene is on Cu. Both spectra are obtained on the same graphene crystal (**b**) after transfer on SiO_2_.

Raman spectroscopy gives quantitative information on the quality of graphene. It allows to quantify the presence of structural defects, the level of graphene doping following the transfer and the evolution of stresses in the graphene layer. The 473 nm laser was focused to a spot size of ~1 µm with a power of ~2 mW and ~0.1 mW respectively for measurement on Cu and SiO_2_. Four samples with and without post-growth annealing were studied and the Raman spectra of one of the samples measured after graphene transfer is depicted on Figure 5b. The presence of the G and 2D peaks and the absence of the D peak confirms the good quality of the transferred graphene. Moreover, Raman measurements were carried out in different areas of the graphene crystal, leading to an average value of the intensity ratio I_2D_/I_G_ of ~3. This confirms that the graphene is a single monolayer and that it remains homogeneous after transfer. The full width at half maximum (FWHM) is ~29 cm^−1^, which emphasizes the high quality of the crystal.

The positions of the G and 2D peaks are compared in Table 1, before transfer (on Cu or on Cu oxide) and after (see Figure 5). The shift of the peaks can be directly linked to the strain and doping induced by the substrate [30].

### 3.4. Strain and Doping Profiles of Graphene

**Figure 6:**
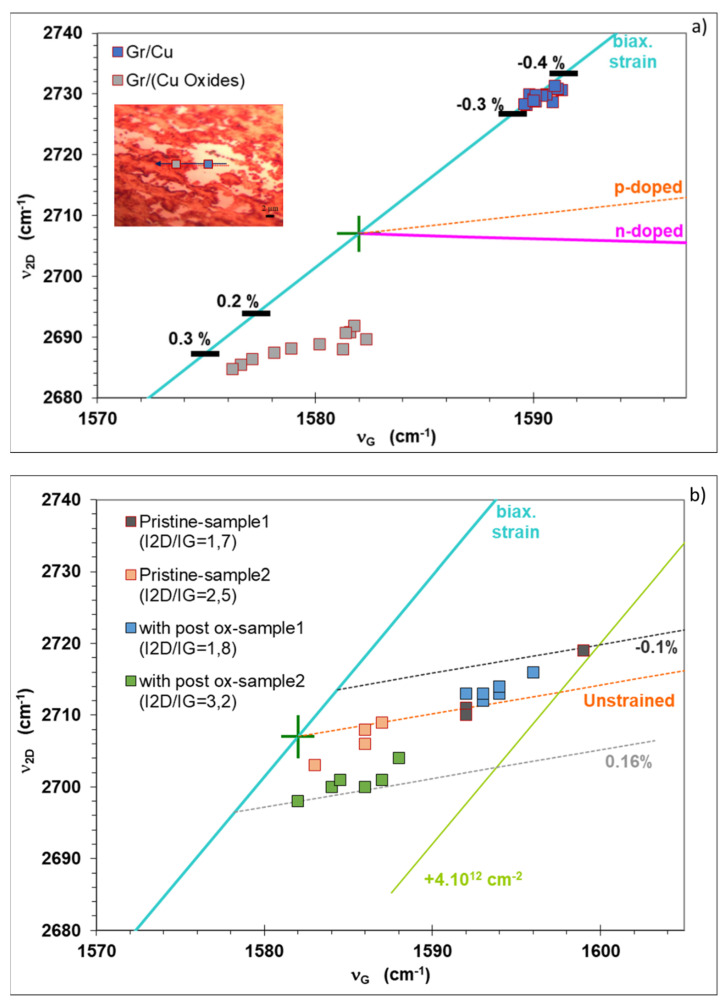
Correlation between the frequencies of the G and 2D Raman peaks of graphene (ν_G_, ν_2D_), from Raman spectra of 4 samples, with and without post-growth annealing. The green cross represents (ν_G0_, ν_2D0_) for undoped and unstrained graphene. The blue line (biax. strain) represents an estimation of (ν_G_, ν_2D_) for undoped graphene under biaxial strain, while the orange (respectively pink) dashed line corresponds to unstrained p-doped (n-doped) graphene. Horizontal black lines in panel a refer to the values of biaxial strain. (**a**) before transfer, (**b**) after transfer, the green line parallel to the blue one represents the (ν_G_, ν_2D_) strain dependency for a p-type doping of 4 × 10^12^ cm^−2^.

This study is based on a vector decomposition method developed to study the influence of strain and doping on graphene as described in references [30,31]. The variation in strain is studied before and after the transfer of graphene, considering the graphene on Cu and graphene on Cu oxide. Since the oxidation of Cu under graphene is not fully homogeneous, as described above (Section 3.1 and Section 3.2), the Raman mapping is performed on two areas, those with “graphene on copper” and “graphene on copper oxide” as illustrated in the inset of Figure 6a. The Supporting Figure S7 shows the peak maxima ν_G_ and ν_2D_, the integrated intensities I_G_, I_2D_ and the intensity ratio I_2D_/I_G_ as a function of position.

We have represented the frequency of the 2D peak as a function of the frequency of the G peak as shown in Figure 6. The green cross in Figure 6 represents the Raman peaks of undoped and unstrained graphene (ν_G0_ = 1582 cm^−1^, ν_2D0_ = 2707 cm^−1^) using an excitation laser at 473 nm. The blue line (biax. strain) represents a prediction of (ν_G_, ν_2D_) for an undoped graphene under biaxial stress. The orange line (p-doped) and the pink line (n-doped) represent predictions of (ν_G_, ν_2D_) for strain-free graphene under p or n charge doping respectively.

The Raman analysis was performed on several graphene samples before transfer showing similar results, one of them is presented in (Figure 6a). The graphene laying on Cu in Figure 6a is under biaxial compressive strain (−0.34 ± 0.03%), whereas the graphene on Cu oxide is subjected to a biaxial tensile strain of (0.3 ± 0.1%).

The Raman analysis was also done for four samples after graphene transfer (on Si/SiO_2_) as shown in Figure 6b. Two samples have been subjected to Cu oxidation by annealing in air after growth of graphene (“with post ox-sample1” and “with post ox-sample2”); and two additional samples without any post-growth treatment (“Pristine-sample1” and “Pristine-sample2”) were measured. The Raman analysis alone is not sufficient to identify the type of doping (n or p). The literature suggests that graphene transferred on SiO_2_ is p-doped [32], and hence, it is reasonable to assume that our samples are p-doped as well. Raman measurements have been obtained on different zones for each sample. The data (illustrated in Figure 6b) shows an evolution close to the orange line which represents a prediction of (ν_G_, ν_2D_) for strain-free graphene with p doping in the range 0 < p < 4 × 10^12^ cm^−2^. Moreover, it can be seen that the graphene biaxial strain is almost completely relaxed after transfer onto Si/SiO_2_ as shown in Figure 6b: the residual strain stands between −0.1% and 0.16% for all the samples [33]. The obtained results are in good agreement with the reported literature [32], which shows that the monolayer graphene having a ratio 1 < I_2D_/I_G_ < 2 is highly doped.

### 3.5. Electrical Characterization of Single-Crystal Graphene after Transfer

**Figure 7:**
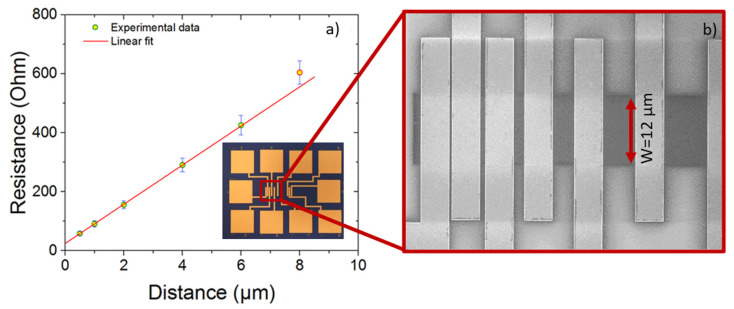
(**a**) Plot of resistance with TLM structure for a graphene width of 12 µm, inset is an optical image of a TLM structure (**b**) SEM image showing a magnification of the framed area in the inset.

The TLM method was used to characterize the contact resistance as well as the sheet resistance of the graphene layer. The optical image of the device fabricated to measure the contact resistance is shown in the inset of Figure 7a. The TLM structure is fabricated on high resistivity silicon substrate, using e-beam lithography and lift-off process with Ni/Au metals contacts. Fabricated samples without annealing are measured and the obtained I-V curves from six TLM patterns shows ohmic contact behavior. The resistances are then extracted and plotted as a function of the separation distance. Variations in measured resistance are proportional to the separation distance between contacts and indicates the homogeneity of the graphene. Thus, we obtained a contact resistance RcW = 150 Ωµm using Ni/Au contacts (with respective thickness of 1.5 nm and 50 nm), and a sheet resistance ρ_sh_ = 797 Ω/square. The microscale sheet resistance measured from devices here are comparable to that of the large area sheet resistance of similarly synthesized and fabricated devices across mm scales, and reflect the high electronic quality of the graphene crystals [34].

## 4. Conclusions

Large graphene single crystals up to a few millimeters were synthesized on Cu substrates, and a reliable graphene transfer method (“controlled bubbling transfer”) has been developed. The applied potential and controlled generation of hydrogen bubbles play an important role in this electrochemical delamination approach. This transfer method is faster than the Cu wet chemical etching method (a few minutes versus a few hours). The quality of graphene is verified by different comparative pre- and post-transfer characterization approaches, including electrical, SEM and Raman spectroscopy.

We have also studied the impact of an extra post-growth annealing step on the Cu oxidation on the large single-crystal graphene. The Cu oxidation is non-uniform under the graphene crystals. The graphene on oxidized Cu is under biaxial tension, whereas the areas where graphene is laying on non-oxidized Cu exhibits biaxial compressive strain with a low doping. Moreover, after graphene was transferred to a host substrate, the strain was strongly reduced, and the graphene doping can vary depending on the host substrate. The electrical characterization of devices on a graphene single crystal shows a sheet resistance of ρ_sh_ = 797 Ω/square and a contact resistance of 150 Ω.µm with Ni/Au contacts. Thus, the electrochemical delamination transfer process paves a path to easy and fast transfer of graphene maintaining its quality and its electrical properties.

## Figures and Tables

**Table 1:** Comparison of the positions of graphene’s G and 2D peaks before and after transfer, from a Raman map of a graphene single crystal obtained from Figure 6.

	Before Transfer	Before Transfer	After Transfer
Graphene Peaks	Graphene on copper	Graphene on copper oxide	Graphene on Si/SiO_2_
ν_G_ (cm^−1^)	1591 ± 2	1579 ± 3	1590 ± 8
ν_2D_ (cm^−1^)	2730 ± 2	2689 ± 3	2708 ± 10

## Supplementary Materials

The following are available online at https://www.mdpi.com/article/10.3390/nano11102528/s1, Figure S1: (a) Optical image of the Cu foil folded in half, the remaining three sides being carefully crimped to obtain an enclosed pocket (b) Illustration of the two-step growth process of the graphene monocrystals. Figure S2: Unfolded Cu pocket, inside face, after growing graphene monocrystals (a) optical image of the Cu foil without flashlight and (b) with flashlight that helps to visualize the large crystals grown on the top with naked eye. Figure S3: Optical image of graphene nanocrystals on Cu foil after post-growth annealing. The resulting oxidation of Cu makes individual crystals visible by eyes. Figure S4: Procedure of graphene transfer to a host substrate using the electrochemical delamination transfer method. Figure S6I: Effect of the applied voltage on H_2_ bubbles generated during transfer (a) vigorous flow for 5 V (b) fewer bubbles for 4 V (c) almost no bubbles for 3 V and (d) no bubbles but no reaction at 2 V. Figure S6II: transfer using optimal parameters (2.7 V, 42 mmol/L) showing the PMMA/Graphene film (a) during the separation from Cu (b) on Cu (c) on SiO_2_ before annealing (d) on SiO_2_ after annealing. Figure S7: the peak maxima ν_G_ (a) and ν_2D_ (b), the integrated intensities IG (c), I2D (d) and the intensity ratio I2D/IG (e) are plotted as a function of position. The corresponding measurement ν_2D_(ν_G_) is shown in Figure 6a of the main text. The image of the sample is shown in (f), where the black line shows the analysed area. Please note that the horizontal axis is oriented from right to left, the zero position approximately corresponding to the limit between Cu (on the right side, x < 0) and Cu oxide (on the left side, x > 0).
