# Supplementary material for: Physical and Electrical Characterization of Synthesized Millimeter Size Single Crystal Graphene, Using Controlled Bubbling Transfer
Source: Nanomaterials (Basel). 2021 Sep 27;11(10):2528. doi: 10.3390/nano11102528 (PMC8541598; doi:10.3390/nano11102528)
Supplement: Supplementary file 1 [file nanomaterials-11-02528-s001.zip › nanomaterials-1337304-proof-SI-final check-risa.pdf]

Supporting Information

Physical and Electrical Characterization of Synthesized Millimeter Size Single Crystal Graphene, Using Controlled Bubbling Transfer

Soukaina Ben Salk [1], Reetu Raj Pandey [1], Phi HQ Pham [2], Di Zhou [1], Wei Wei [1], Guillaume Cochez [1], Dominique Vignaud [1], Emiliano Pallecchi [1,*], Peter J Burke [2] and Henri Happy [1,*]

[1] University of Lille, CNRS, UMR 8520 - IEMN – Institut d'Electronique de Microélectronique et de Nanotechnologie, F-59000 Lille, France; Soukaina.ben-salk@univ-lille.fr (S.B.); reetu-raj.panday@univ-lille.fr (R.R.P.); di.zhou@univ-lille.fr (D.Z.); wei.wei@univ-lille.fr (W.W.); guillaume.cochez@univ-lille.fr (G.C.); dominique.vignaud@univ-lille.fr (D.V.)

[2] Department of Electrical Engineering and Computer Science, University of California, Irvine, CA 92697, USA; PhiP1@uci.edu (P.H.P.); pburke@uci.edu (P.J.B.)

* Correspondence: emiliano.pallecchi@univ-lille.fr (E.P.); henri.happy@univ-lille.fr (H.H.)

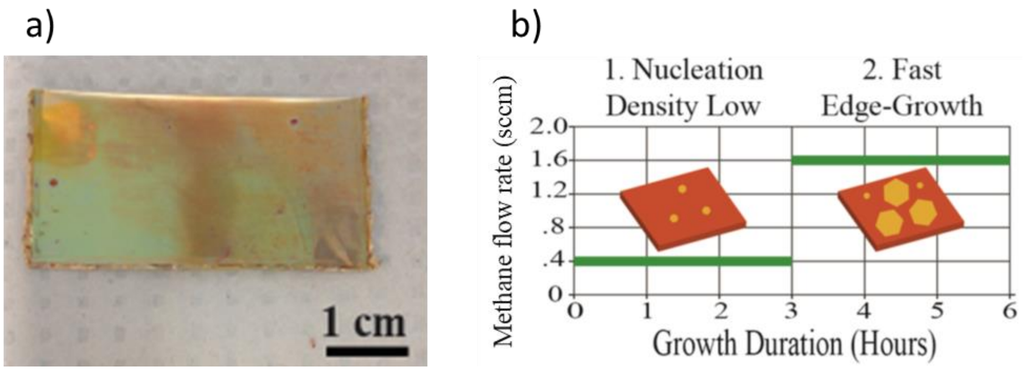

Figure S1: (a) Optical image of the Cu foil folded in half, the remaining three sides being carefully crimped to obtain an enclosed pocket (b) Illustration of the two-step growth process of the graphene monocrystals.

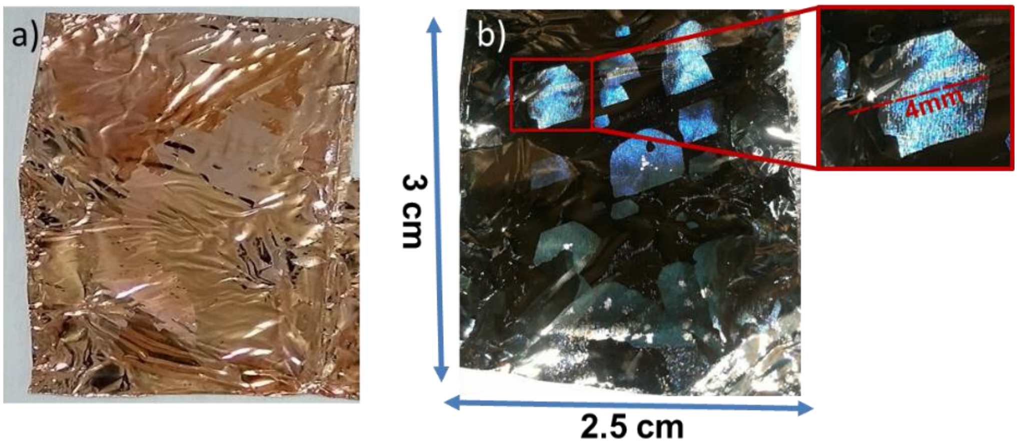

Figure S2: Unfolded Cu pocket, inside face, after growing graphene monocrystals (a) optical image of the Cu foil without flashlight and (b) with flashlight that helps to visualize the large crystals grown on the top with naked eye.

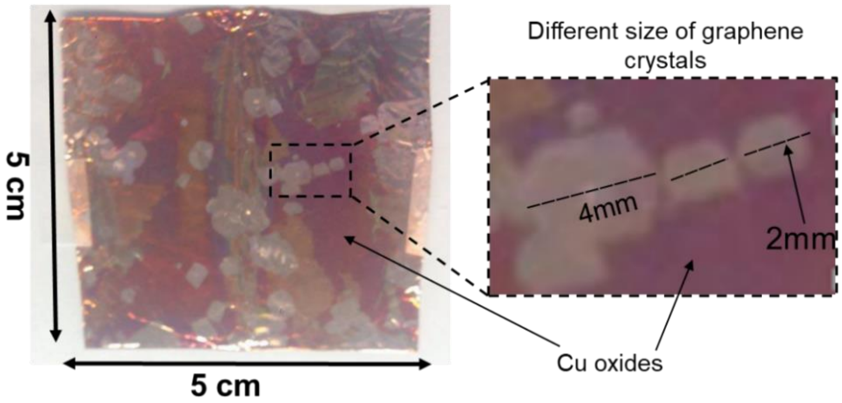

Figure S3: Optical image of graphene nanocrystals on Cu foil after post-growth annealing. The resulting oxidation of Cu makes individual crystals visible by eyes.

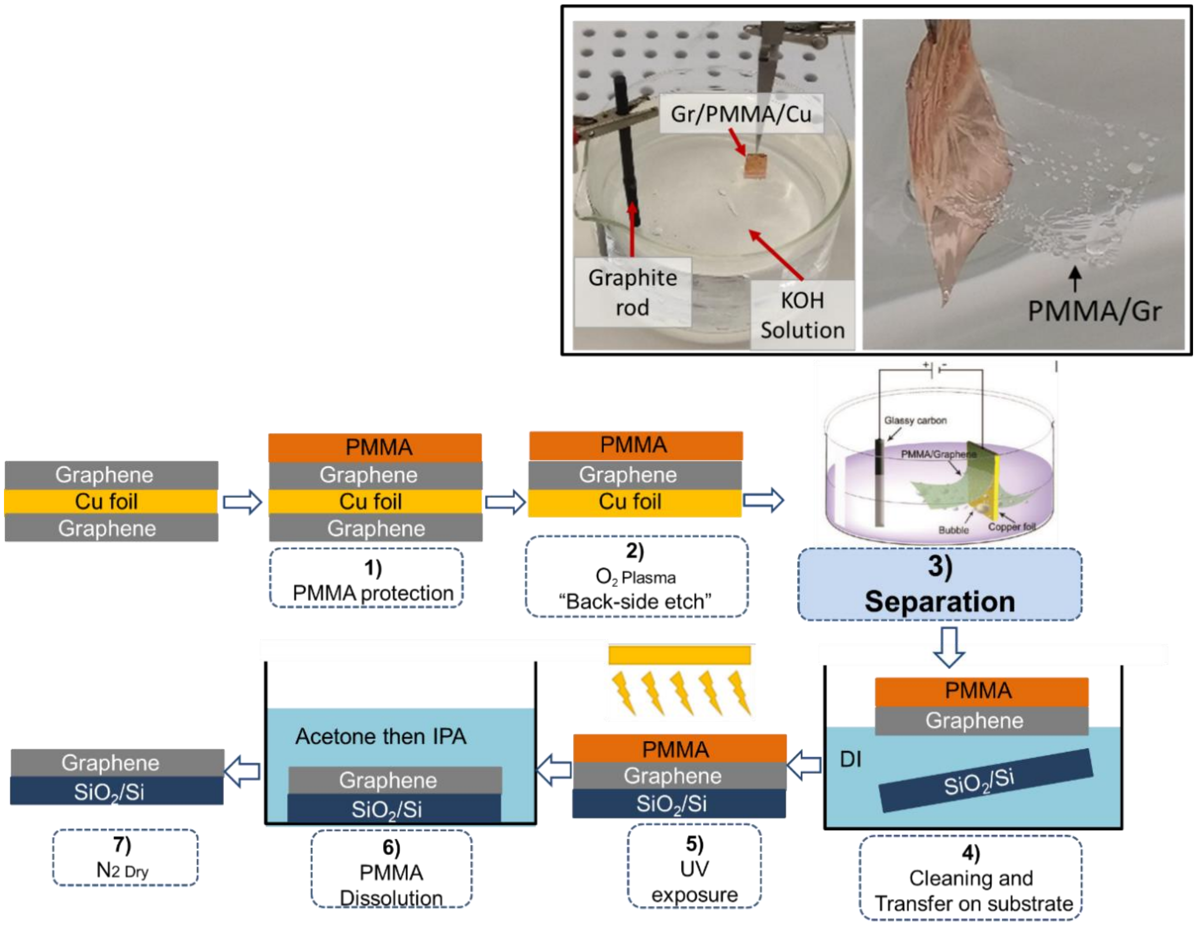

Figure S4: Procedure of graphene transfer to a host substrate using the electrochemical delamination transfer method.

The electrochemical delamination transfer method consists of seven steps as described below:

Step 1: Protection of graphene with a layer of PMMA

The synthesized graphene on Cu foil is spincoated with PMMA 5% 950K.

Step 2: Etching of graphene on the backside of the Cu foil by oxygen plasma

O2 Plasma etching parameters: 50W, 100mTorr, 25sccm. (Reactive Ion Etching)

Step 3: Electrochemical delamination of graphene from the Cu foil (see details in the next paragraph)

Step 4: Graphene cleaning and transfer

After separating graphene from growth substrate, the film PMMA/Graphene is floating on the solution surface. It is then rinsed in dionised water many times then transferred on Si/SiO2 substrate. In order to remove water at the interface between graphene and the substrate, the sample is baked at 90°C.

Step 5: Exposure of the sample to UV light

Deep UV during 30 min at 500W in order to facilitate PMMA removal as well as its residues.

Step 6: Dissolution of PMMA

Acetone bath for 30 min at 30-35°C followed by IPA bath.

Step 7: Drying gently with nitrogen flow

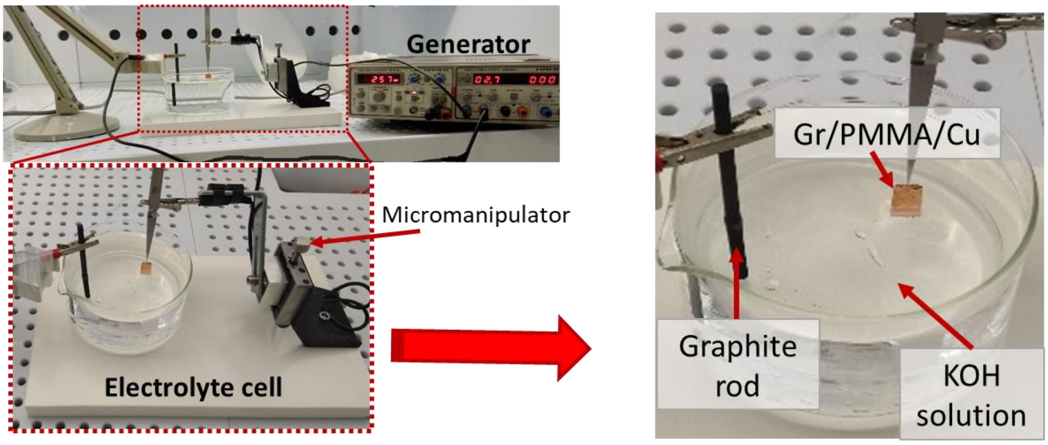

Figure S5: Experimental setup for electrochemical delamination of graphene.

Optimization of the delamination process

We first looked for the optimal voltage allowing the separation of graphene from the Cu foil while generating the minimum amount of hydrogen bubbles. The distance between the electrodes was fixed (7 cm), using a clean grassy carbon counter electrode, and a standard electrolyte solution (0.2 mol/L KOH). We started with the higher applied bias and gradually went to lower bias. When the applied bias was higher than 4V, vigorous hydrogen bubbles were appearing which causes the graphene/PMMA film to roll on itself during separation. By decreasing the applied bias, the separation of graphene from Cu foil is still observed when the applied bias was 3V (Figure S6-Ic), but no reaction takes place when applied bias was 2V Figure S6-Id. By empirically varying the voltage value between 2V and 3V until the graphene begins to detach from the Cu, we concluded that 2.7V is the minimum voltage that allows separation of graphene from Cu while generating the minimum amount of hydrogen bubbles in our system.

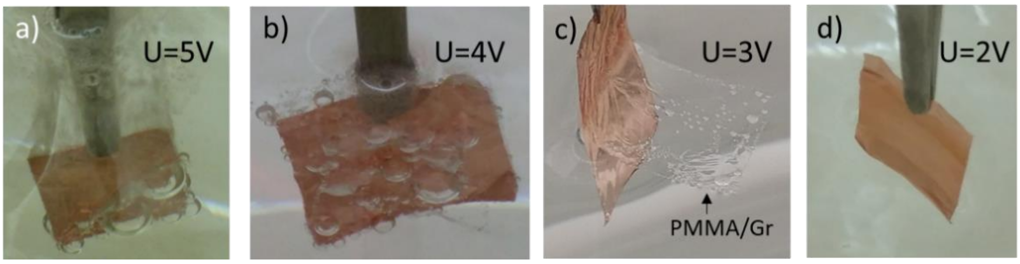

Figure S6-I: Effect of the applied voltage on H_2 bubbles generated during transfer (a) vigorous flow for 5V (b) fewer bubbles for 4V (c) almost no bubbles for 3V and (d) no bubbles but no reaction at 2V.

Considering the optimal obtained voltage (2.7V) for graphene separation from the Cu substrate, next we varied the concentration of the electrolyte. In a volume of 500 ml deionized water (DI), the KOH solution (40%) was added by amounts of 0.5 ml (the concentration of the stock solution is 7140 mmol/L) until graphene begins to detach from Cu. An optimum of 42 mmol/L (3ml of KOH (40%) in 500ml of DI) is obtained for a 2.7V allowing a transfer without defects and with the minimum contamination. The figure S6-IIa shows the PMMA / graphene film floating on the surface of the solution without the presence of bubbles trapped under the film. The same film is shown just after transfer on SiO_2 (c) and after annealing (d). The film is homogeneous and does not feature any folds or tears.

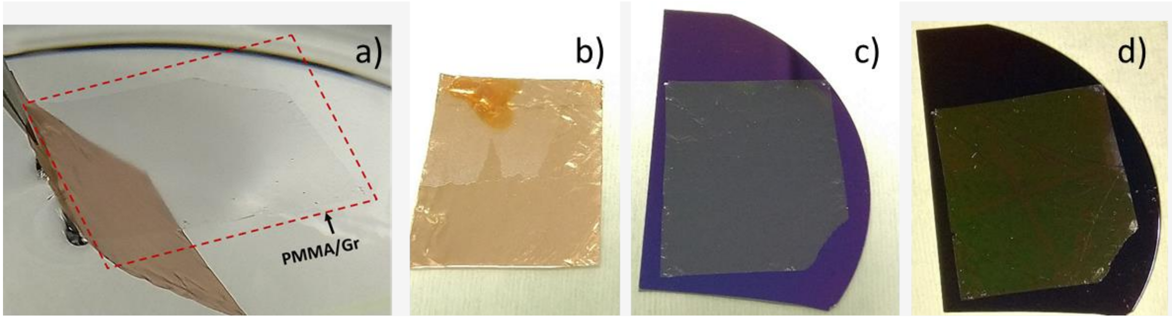

Figure S6-II: transfer using optimal parameters (2.7 V, 42 mmol/L) showing the PMMA/Graphene film (a) during the separation from Cu (b) on Cu (c) on SiO2 before annealing (d) on SiO2 after annealing.

Raman mapping characterization

The $\nu_{2D}(\nu_G)$ curve obtained during a 1D map measurement is shown in figure 6a. The corresponding characteristics of the G and 2D Raman peaks (peak position, integrated intensity and integrated intensity ratio) are shown in figure S7, now as a function of position on the sample. They show the clear dependency of these measurements on the graphene supporting substrate, either Cu or Cu oxide.

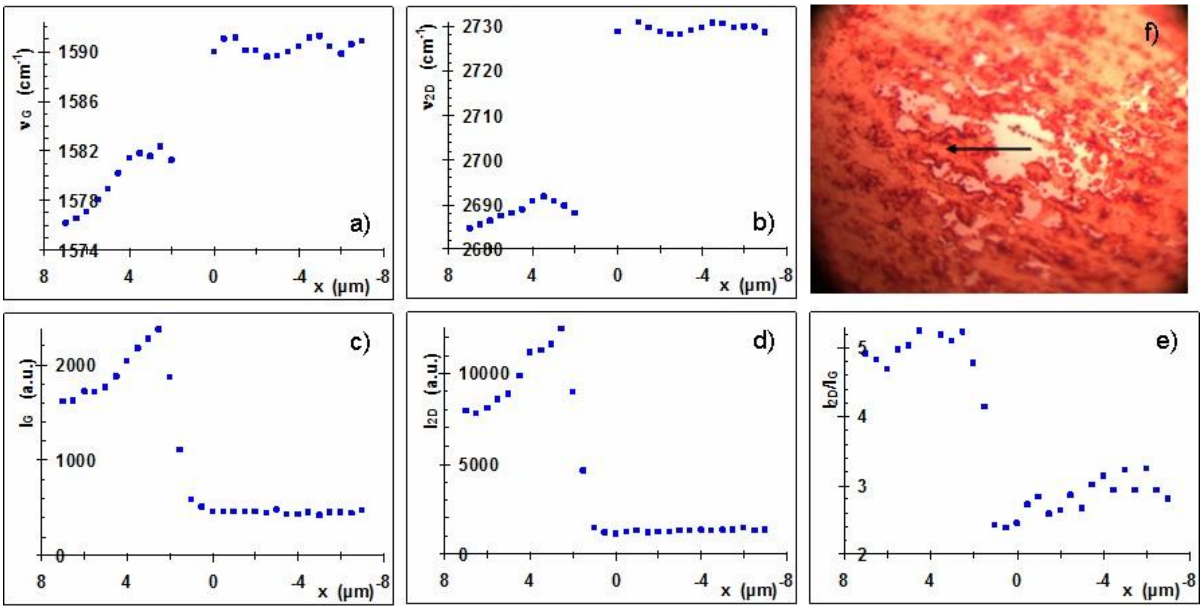

Figure S7: The peak maxima ν_G (a) and ν_{2D} (b), the integrated intensities I_G (c), I_{2D} (d) and the intensity ratio I_{2D}/I_G (e) are plotted as a function of position. The corresponding measurement $\nu_{2D}(\nu_G)$ is shown in figure 6a of the main text. The image of the sample is shown in (f), where the black line shows the analysed area. Please note that the horizontal axis is oriented from right to left, the zero position approximately corresponding to the limit between Cu (on the right side, x<0) and Cu oxide (on the left side, x>0).
